# Opisthorchiasis in infant remains from the medieval Zeleniy Yar burial ground of XII-XIII centuries AD

**DOI:** 10.1590/0074-02760150156

**Published:** 2015-12

**Authors:** Sergey Mikhailovich Slepchenko, Alexander Vasilevich Gusev, Sergey Nikolaevich Ivanov, Evgenia Olegovna Svyatova

**Affiliations:** 1 Siberian Branch of the Russian Academy of Sciences, Institute for Problems of the Development of the North, Tyumen, Siberia, Russia; 2 YaNAO Arctic Research Center, Archeology Department, Archeology and Ethnology Sector, Salekhard, YaNAO, Russia; 3 Institution of Culture of Sverdlovsk Region, Center for Protection and Use of Monuments of History and Culture of Sverdlovsk Region, Scientific and Production Center, Ekaterinburg, Urais, Russia

**Keywords:** Opisthorchis felineus, paleoparasitology, paleopathology, Western Siberia, health status, food processing

## Abstract

We present a paleoparasitological analysis of the medieval Zeleniy Yar burial ground of the XII-XII centuries AD located in the northern part of Western Siberia. Parasite eggs, identified as eggs of *Opisthorchis felineus*, were found in the samples from the pelvic area of a one year old infant buried at the site. Presence of these eggs in the soil samples from the infant’s abdomen suggests that he/she was infected with opisthorchiasis and imply consumption of undercooked fish. Ethnographic records collected among the population of the northern part of Western Siberia reveal numerous cases of feeding raw fish to their children. Zeleniy Yar case of opisthorchiasis suggests that this dietary custom has persisted from at least medieval times.

Paleoparasitological analysis can be carried out using a wide range of archaeological and biological objects, including samples from the pelvic/abdominal areas, coprolites, hair, mummified tissues, and even skeletal remains (Araújo & Ferreira 1996, Bouchet 1997,Le Bailly et al. 2005, Araújo et al. 2008). Identification of endoparasites can complement archaeological data on the paleodiet, subsistence practices, and food processing techniques, as well as trace migration patterns and help to reconstruct community health of prehistoric populations (Reinhard 1988, 19,1992a, b, Araújo & Ferreira 1996, 20,^2000^, Horne & Tuck 1996, Bouchet et al. 2001,Reinhard & Araújo 2008, Jaeger et al. 2013, Slepchenko & Ivanov 2015, Yeh et al. 2015).

Paleoparasitological research is still nascent in Russia. Arkadiy Savinetsky and Aleksandr Khrustalev in 2013 presented a meta-analysis of endoparasites found in the animal faecal depositions from caves with archaeological deposits dating from as early as 40,000 years ago to XX century. Their study identified helminth eggs belonging to*Nematodirus* sp., *Oxyuris*(*Oxyurida*), *Fasciola* sp.,*Dicrocoelium* sp., *Trichuris*, *Capillaria*sp., *Diphyllobothrium* sp., *Opisthorchis felineus*, *Alaria alata*, and *Dioctophyma renale*. The diversity of helminths in the animal faeces provided insights into the subsistence practices of the early humans and hazards of animal farming in those regions.

Paleoparasitological analysis of dog coprolites from Maray I settlement (dating to 2645 ± 30 BP), located in the forest-steppe zone of West Siberia, revealed larvae of*Strongyloides papillosus*, *Strongyloides westeri*, and *Strongyloides stercoralis* (Zach et al. 2011, Tsembalyuk 2013), suggesting heavy parasitic loads in the human settlements of the region. Recent analysis of the XVII century soil and dog coprolites samples from Mangazeya, the earliest Russian town located beyond the Arctic Circle in West Siberia. The eggs of *O. felineus*, *Diphillobathrium latum*, *Trichocephalus*sp., *Toxocara canis*, and *Fasciola hepatica*were identified there (Vizgalov et al. 2013).

The only paleoparasitological investigation of the recent indigenous populations from Western Siberia was carried out on the samples from the pelvic area from the XVII-XIX centuries Selkup Kikki-Akki burial site. Tapeworm eggs of *Diphyllobothrium*sp., a typical fish endoparasite, showed that uncooked or undercooked fish served as the primary source of intestinal parasites in Selkup communities (Slepchenko & Ivanov 2015). This finding corroborated the ethnographic records that the Selkups consumed large quantities of raw or minimally processed fish (Khomich et al. 2002,Tuchkova 2013). The absence of *O. felineus*, a fish endoparasite common in Ob and Irtysh river basins, in samples from the Kikki-Akki burial ground suggested that Selkups from Kikki-Akki rarely migrated to the Ob and Irtysh region for fishing (Yossepowitch et al. 2004, Bonina & Fedorov 2010, Mordvinov et al. 2012). Thus, the Selkups migration routes were probably limited to the nearby Taz River Basin and, perhaps, the Yenisei and the Pur river basins which lack*Opisthorchis* (Slepchenko & Ivanov 2015).

Here, we present an analysis of helminth eggs from the soil samples collected from the pelvic and abdominal area of the child buried in grave 48 (hereafter M48) of the Zeleniy Yar cemetery dated to between XII-XIII centuries. The Zeleniy Yar archaeological site is located in the Cisuralian area of Yamalo-Nenets Autonomous Okrug in the north of Western Siberia (Fig. 1). It is situated on a flood plain island between the Poluy River and the Gorny Poluy anabranch. Remains of an early metallurgy workshop, including two melting furnaces dating to VI-VII centuries AD, have been recovered from the site. Two associated burial grounds have been dated to the VIII-IX centuries and the XII-XIII centuries.

**Fig. 1:**
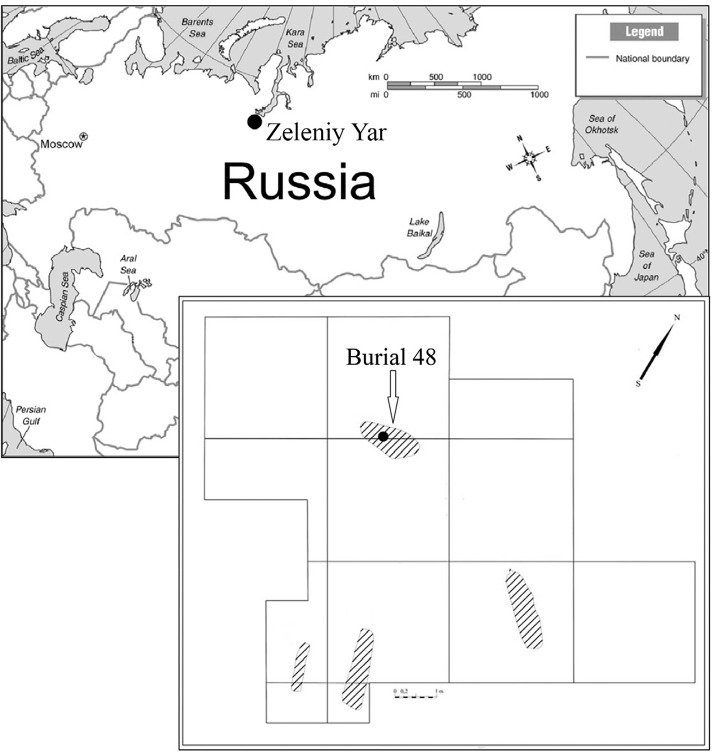
: location the archaeological site Zeleniy Yar. Distribution of burials on the archaeological site Zeleniy Yar in 2014. The black point is marked the place of sampling in the burial 48.

## MATERIALS AND METHODS

The site was excavated by the authors during the 2014 field season. Four burials dating from the XII-XIII centuries were excavated and analysed at the same time. Samples were collected from M48, since it was the only burial that remained intact and undisturbed by carnivores.

Burial M48 contained remains of an infant with an estimated age around one year, based on dental eruption and skeletal development. The body was in supine position with the head placed at a northwest orientation. The infant’s arms were stretched along the body with the palms positioned on the thighs. The neck was bent, so that the chin was pressed to the chest, suggesting that there was some support made from perishable material was placed under the head. The infant was wrapped in a fur garment adorned with copper plates, which was further wrapped in a hide with fur and a layer of birch bark (Gusev 2015).

**Fig. 2:**
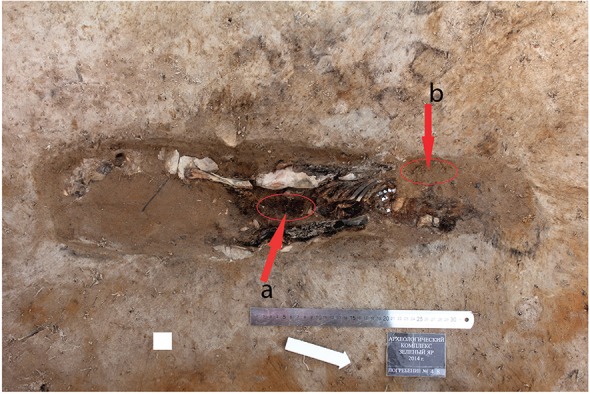
: infant skeleton was excavated at Zeleniy Yar and place of sampling. a: the place of abdomen where the soil sample was collected for the paleoparasitological study. b: the place where the control soil sample was collected.

Since the infant’s sacrum, the os coxae and lumbar vertebra were completely decomposed, the sample was collected from the abdominal region (Fig. 2). The sample weighed about 30 g and was vacuum-packed in the field. In addition, a 50 g control sample was taken from the vicinity of infant’s head at the same depth as the abdominal sample.

In the laboratory, 3% sodium hydroxide solution was added to the dry sample (Fry 1985, Dufour & Le Bailly 2013). In an hour and a half, the liquid was sifted through a sieve, filled into a plastic test tube, and centrifuged for 7 min (1,500 revolutions per min); distilled water was added to achieve neutral pH level. Upon completing this cleaning procedure, we added a rich sodium nitrate solution (1.38-1.40 g/cm^3^) to the residue. Sample separation was performed in the same centrifugal tubes. After multiple centrifuge cycles with 0.5% Na_3_PO_4_ aqueous solution and glycerin at a temperature of 22 degrees, we gathered the supernatant fraction. Having just a few organic particles at hand, we still managed to prepare 20 microslides, following the recommended standard methods (Reinhard et al. 1986, Araújo et al. 1998). Microscopic examination was conducted using AxioSkop 40 and MicMed 2 var.2. microscopes under 80X and 400X magnification. Measurements were obtained using AxioVision 4.6 and Scope Photo 3.0 software.

## RESULTS

**Fig. 3:**
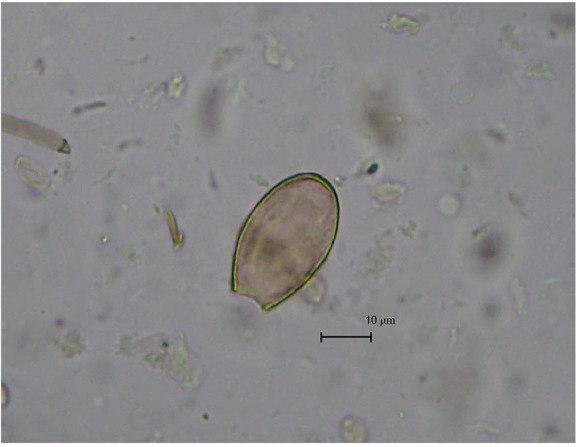
: eggs suggestive of *Opisthorchis felineus* found in infant remains from the medieval Zeleniy Yar burial ground of XII-XIII centuries AD.

The microscopic examination of the slides revealed four helminth eggs of an oval shape and of light yellow colour. The operculums of eggs were absent. Some eggs had slight shoulders at the area of operculum attachment. At the pole opposite to operculum, there was a knob. The eggs measured 34.25-32.39 μm in length and 24.5-18.01 μm in width. Based on their morphology and size, these eggs belong to the trematodes group of *O. felineus* (Fig. 3). The control sample was free of eggs. Epidemiologic records indicate a high incidence of opisthorchiasis among the modern-day populations of the area where the Zeleniy Yar burial ground is located (Istomin et al. 2003), corroborating our interpretation.

## DISCUSSION

*Differential diagnosis* - In order to identify species affiliation of the eggs, we performed a differential diagnosis. The eggs found in the samples from the burial may belong to several different genera of helminths. Morphological characteristics such as an oval shape, light yellow colour, size, and knob have to be further analysed with the aid of differential diagnosis which involves comparing helminth eggs of the cestodes *Diphyllobothrium* sp. and of the trematodes *Clonorchis sinensis*, *Opisthorchis viverrini*, *Metorchis bilis.*

Diphyllobothriasis is widespread throughout Western Siberia. The Cisuralian area of Yamalo-Nenets Autonomous Okrug (with Zeleniy Yar burial ground there) has the highest incidence of this helminth infection among humans in the region. Some records show 340.3 cases per 100,000 people (Rospotrebnadzor State Reports 2011). In the Ural Mountain area and Eastern Siberia, *D. latum* is the most common fish tape worm infecting humans. In Western Siberia *Diphyllobothriumdendriticum*and *Diphyllobothriumditremum* are also common in humans, although the epidemiological impact of the latter on the local human populations is minimal (Yastrebov 2013).

Having the same colour, elliptic shape, and knob, *Diphyllobothrium*sp. eggs look superficially similar to the eggs of*Opisthorchis*. Nevertheless, we excluded the tapeworm eggs as the infection source, because of their bigger size. Typical*Diphyllobothrium* eggs measure 58-75 μm x 40-50 μm (Ash & Orihel 2007), considerably larger than the 24.5-18.01 μm eggs reported here.

Trematodes, *C.sinensis* and *O.viverrini,* also produce eggs that are morphologically very similar to the observed eggs (Kaewkes et al. 1991, Zhou et al. 2007). Their size falls within the same range as that of the eggs we have found (29 μm x 17 μm and 27 μm x 15 μm, respectively). Eggs of trematodes are ovoid-shaped, longish, yellow-brown, and have shoulders at the place of an operculum attachment and a knob at the opposite egg pole (Sadun 1955, Liu & Chen 1998). Therefore, excluding *C. sinensis* and*O. viverrini* is difficult. However, the epidemiologic records show that the trematodes *C. sinensis* and *O.viverrini*are unlikely to be the source of infection. Although both helminths are present in Southeast Asia, Korea, and China (Posokhov 2004, Lun et al. 2005,Sripa et al. 2007), they are nonexistent in Western Siberia (Posokhov 2004). The only known case of clonorchiasis in Russia has been documented in the Amur River Basin. Therefore, despite the morphological similarity to the recovered eggs, the trematodes *C.sinensis* and *O.viverrini* are unlikely to be the source of the infection, albeit these cannot be completely ruled out.

The eggs present in the M48 sample are also morphologically similar to eggs of*M. bilis*, a helminth from family Opisthorchidae that is present in Western Siberia. Since eggs of *O. felineus* and *M. bilis* cannot be differentiated by any common medical and parasitological methods, it is likely that some of the diagnosed opisthorchiasis infection in Western Siberia is caused by metorchosis infection or by both (Skrjabin & Petrov 1950, Sidorov 1983, Romashov et al. 2005). However, genus *Metorchis* is more commonly present in the southern territory of Western Siberia, while *O. felineus* has been recorded in its northern part (Fattakhov 1996). Therefore, final animal hosts and humans are predominantly infected with opisthorchiasis in the northern part of Western Siberia, with its highest rate in the Middle Ob Area, whilst metorchosis infection rate increases towards the southern regions of Siberia (Fattakhov 1996). A very low incidence of metorchosis in the lower course of the Ob River, where Zeleniy Yar burial ground is located, has been documented. This low occurrence of metorchosis can be explained by a low frequency of*Bethynia tentaculata* (family Bithyniidae, genus*Bethynia*) molluscs that serve as the principal first intermediate host for metorchosis. Although *Codiella inflate* (family Bithyniidae, genus *Bethynia*), the secondary intermediate host of metorchosis, is present in the region, this mollusc has a low susceptibility of metorchosis, limiting its propagation (Fattakhov 1996).

A high frequency of opistarchosis infection is recorded among the modern-day populations of the Cisuralian area of Yamalo-Nenets Autonomous Okrug. In 2003, the prevalence rate was 2,585 cases per 100,000 people (Istomin et al. 2003). Among children under the age of 14, opistarchosis was present in 110 individuals out of 100,000 (Rospotrebnadzor State Reports 2011). For comparison, the prevalence rate for opistarchosis in the Ob River tributaries is 10-104 cases per 1,000 people (Karpenko et al. 2008). Up to 100% of the indigenous people, among Khanty, Mansi, and Nenets ethnic communities of the Ob and Irtysh basins in West Siberia are infected with these helminths (Schustov et al. 2002). Such a high incidence of opistarchosis among indigenous people of the reason is caused by the heavy parasitic loads found in fish of Ob and Irtysh rivers. In the middle and the lower Ob River Basin, the opistarchosis infection rate in *Leuciscus idus* and*Leuciscus icuciscus* fish is between 80-100%. The invasion degree is 4.5 and 7.5 larvae per gram of muscles, for *L. idus* and*L. Icuciscus*, respectively [Fattakhov (1996) (p. 14)]. Moreover, the high frequency of opistarchosis among the local people is caused by the consumption of raw, undercooked, or slightly salted fish (Istomin et al. 2003).

Thus, we may infer that the helminth eggs in the samples from the Zeleniy Yar burial ground most likely belonged to the fish tapeworm genus *O. felineus.*The presence of *Opisthorchis* eggs in the samples from the burial of an infant under one is noteworthy, as it implies early introduction of solid foods into infant diet. The infant could be infected with opisthorchiasis only by consuming raw or minimally processed contaminated fish. The incubation period of opisthorchiasis is approximately three-four weeks after the initial infection of the final host (Vozianova 2000). Consequently, we may infer that the infant from M48 was given raw fish as a nursing supplement at least one month before his/her death.

The abundance of fish in the rivers of Western Siberia determined the local subsistence strategies at least since the terminal Neolithic (Tupakhina 2013). Large quantities of fish bones were recovered from the Iron Age Ust-Poluy archaeological site, located in the lower Ob River Basin, suggesting that fish was an important and possibly the main food source at the time. Similarly, the medieval Zeleniy Yar archaeological site is also rich in fish deposits (Aleksashenko et al. 2005).

According to ethnographic reports (Bartenev 1896, Dunin-Gorkavich 1910), the indigenous people of the northern part of Western Siberia consumed large quantities of raw fish. In 1715, the Russian ethnographer Gregory Novitsky recorded that the population of the Ob River Basin consumed fish, including fish intestines, in large amounts (Zuev 1947, Novitsky 1989). People ate considerable quantities of fish liver oil, a local specialty food. Diet included fresh raw fish in the summer and frozen raw fish in the winter. The same subsistence practice was described by ethnographer Vasily Zuev in 1771-1772 (Zuev 1947). An XVIII-century historian Gerhard Miller (1742-1746) wrote about the practice of feeding raw fish to young children among Nents. Nents children over one year old were given deer oil and chopped fish in order to get them accustomed to eating raw food (Miller 1999, 2000, Ehlert 2006). Likewise, the Ostyaks (also known as Khanty) fed adult food to children. Furthermore, Russian historian Constantine Nosilov recorded consumption of several fish species, including *Stenodus leucichthys*, *L. idus*, *Rutilus rutilus*, *Lota maculosa*, *Esox lucius*, *Perca fluviatilis*, *Gymnocephalus* by Khanty (Nosilov 1937). Some of these species, such as*L. Idus* and *R. rutilus*, could be carriers of*O. felineus* larvae (WHO 1995), serving as the source of human infection when consumed raw.

The *Opisthorchis* eggs presence in the samples from the infant’s burial in the Zeleniy Yar burial ground reveals a dietary custom of consuming raw or undercooked fish during the Middle Ages in Western Siberia. Opisthorchiasis infection observed in the very young infant indicates early supplementation of breast milk with minimally processed fish. Ethnographic records from the northern part of Western Siberia corroborate these findings, revealing numerous cases of feeding raw fish to their children, which suggests that this dietary practice has considerable antiquity.
